# Dynamic Contrast-Enhanced MRI Assessment of Hyperemic Fractional Microvascular Blood Plasma Volume in Peripheral Arterial Disease: Initial Findings

**DOI:** 10.1371/journal.pone.0037756

**Published:** 2012-05-25

**Authors:** Bas Versluis, Marjolein H. G. Dremmen, Patty J. Nelemans, Joachim E. Wildberger, Geert-Willem Schurink, Tim Leiner, Walter H. Backes

**Affiliations:** 1 Department of Radiology, Maastricht University Medical Center (MUMC+), Maastricht, The Netherlands; 2 Department of Surgery, Maastricht University Medical Center (MUMC+), Maastricht, The Netherlands; 3 Department of Epidemiology, Maastricht University Medical Center (MUMC+), Maastricht, The Netherlands; 4 Cardiovascular Research Institute Maastricht (CARIM), Maastricht, The Netherlands; University of Otago, New Zealand

## Abstract

**Objectives:**

The aim of the current study was to describe a method that assesses the hyperemic microvascular blood plasma volume of the calf musculature. The reversibly albumin binding contrast agent gadofosveset was used in dynamic contrast-enhanced magnetic resonance imaging (DCE MRI) to assess the microvascular status in patients with peripheral arterial disease (PAD) and healthy controls. In addition, the reproducibility of this method in healthy controls was determined.

**Materials and Methods:**

Ten PAD patients with intermittent claudication and 10 healthy control subjects were included. Patients underwent contrast-enhanced MR angiography of the peripheral arteries, followed by one DCE MRI examination of the musculature of the calf. Healthy control subjects were examined twice on different days to determine normative values and the interreader and interscan reproducibility of the technique. The MRI protocol comprised dynamic imaging of contrast agent wash-in under reactive hyperemia conditions of the calf musculature. Using pharmacokinetic modeling the hyperemic fractional microvascular blood plasma volume *(V_p_,* unit: %) of the anterior tibial, gastrocnemius and soleus muscles was calculated.

**Results:**

*V_p_* was significantly lower for all muscle groups in PAD patients (4.3±1.6%, 5.0±3.3% and 6.1±3.6% for anterior tibial, gastrocnemius and soleus muscles, respectively) compared to healthy control subjects (9.1±2.0%, 8.9±1.9% and 9.3±2.1%). Differences in *V_p_* between muscle groups were not significant. The coefficient of variation of *V_p_* varied from 10–14% and 11–16% at interscan and interreader level, respectively.

**Conclusions:**

Using DCE MRI after contrast-enhanced MR angiography with gadofosveset enables reproducible assessment of hyperemic fractional microvascular blood plasma volume of the calf musculature. *V_p_* was lower in PAD patients than in healthy controls, which reflects a promising functional (hemodynamic) biomarker for the microvascular impairment of macrovascular lesions.

## Introduction

Functional measurements in peripheral arterial disease (PAD) are used for diagnostic purposes and for quantifying the hemodynamic consequences of obstructive arterial lesions [1], [2], [3], [4]. Among these functional measurements, the ankle brachial index (ABI) is the most recognized and widely applied test for diagnosis and therapy monitoring in PAD [1], [2], [3], [4], [5], [6], [7], [8], [9]. ABI measurements only represent macrovascular functionality, whereas PAD is known to affect the microcirculation as well, particularly in patients with diabetes [10], [11]. Functional assessment of the microvascular status of the lower extremities could therefore be a valuable addition to the current diagnostic work-up in PAD to objectively determine disease severity and might be useful in evaluation of therapeutic efficacy in PAD [12], [13], [14], [15]. MRI is well-suited for comprehensive diagnostic imaging of both macrovascular morphology of the peripheral vascular tree using contrast-enhanced MRA (CE-MRA) [16], [17], [18] and the microvascular functionality by dynamic contrast-enhanced (DCE) MRI [14], [19], [20], [21].

Commonly used functional measures in DCE MRI are the rate constant (*k*) and the transfer constant (*K^trans^*), which both describe the wash-in of a contrast agent. These measures reflect a combination of the rate of microvascular reactivity and permeability, both of which may be significantly impaired in patients with PAD [14], [19], [22], [23]. The rate of vascular reactivity, as determined by rate constant *k* and transfer constant *K^trans^*, however, decreases with age, mainly due to slow progression of vascular resistance as reflected by an increased intima thickness versus luminal diameter ratio [24]. Therefore, the clinical relevance of these measures in PAD patients is questionable. In mild PAD, i.e. intermittent claudication, symptoms generally arise and progress slowly during exercise (impaired active hyperemia). Critical ischemia, on the other most severe end of the pathophysiological spectrum, is a chronic condition characterized by hypoperfusion of the distal lower extremity at rest. It is conceivable that in both of these conditions, the absolute degree of microvascular dilatation, rather than its rate, is of more clinical relevance and a better reflection of clinical severity of microvascular disease in PAD compared to rate and transfer constants. We hypothesize that maximum microvascular dilatation can be assessed by determining the hyperemic fractional microvascular blood plasma volume (*V_p_*) using DCE MRI. Microvascular blood plasma volume, however, is hard or even impossible to measure using extracellular contrast agents, as clinically applied low-molecular weight (i.e. small-sized) contrast agents extravasate rather rapdily into the interstitial space [22], [25], [26]. Recently introduced blood pool agents are largely prevented from leaking into the interstitial space by a strong, reversible albumin binding and therefore theoretically allow a more reliable assessment of microvascular blood plasma volume with DCE MRI [27], [28].

The main purpose of the current study was to describe a method to assess hyperemic microvascular blood plasma volume of the calf musculature and to investigate its ability to discriminate between PAD patients and healthy control subjects using the blood pool contrast agent gadofosveset in dynamic contrast-enhanced (DCE) MRI. Also, the interreader and interscan reproducibility of this method was determined in healthy control subjects.

## Materials and Methods

### Study Population

Ten patients (age mean ± SD: 66.5±9.9 years; 8 males and 2 females) with proven PAD (intermittent claudication, Fontaine stage II, [5]), diagnosed by the vascular surgeon, and 10 healthy control subjects without signs and symptoms of PAD (age 24.1±2.2 years; 3 males and 7 females) were included in this study. Exclusion criteria were diabetes mellitus, hemodynamic instability, contra-indications for MRI (i.e. claustrophobia, known gadolinium based contrast agent allergy, and estimated glomular filtration rate <30 mL/kg/1.73 m^2^. The institutional medical ethics committee approved the study and all subjects gave written informed consent before inclusion.

### MRI Protocol

An overview of the imaging protocol is given in figure S1. The entire DCE MRI examination lasted approximately 15 minutes. All scans were performed on a 1.5-T commercially available MRI system (Intera, Philips Medical Systems, Best, The Netherlands). For all acquisitions a dedicated 12-element three-station peripheral vascular phased-array coil (4 elements/station) was used. Subjects were imaged in the supine position and care was taken not to deform calf muscle by calf-bed contact. All subjects were lying in this position for at least 30 minutes before the DCE MRI measurement was started. During this time a conventional three-station contrast enhanced MR angiography (CE-MRA) exam was performed in the subject. Therefore, prior to the start of the DCE MRI measurement, all subjects had already received 8 mL of gadofosveset (Ablavar®, Lantheus Medical Imaging, Billerica, MA).

PAD patients were examined once, whereas in healthy control subjects the entire exam was repeated on a different day (interval mean ± SD: 9.3±3.5 days) to determine normative values and the reproducibility of the technique.

#### Survey

A non-enhanced time-of-flight (TOF) scan of the pelvic, upper and lower leg station was acquired to prescribe the imaging volumes of interest for subsequent morphological and functional imaging. A turbo field echo (TFE) pulse sequence was used with a 180° inversion prepulse to suppress stationary tissues. Thirty-one transverse slices per station were acquired with 3.3-mm slice thickness and 11-mm interslice gap, and an inferiorly concatenated saturation band. The standard quadrature body coil was used for signal transmission and reception. For positioning of the 3D angiography volumes, maximum intensity projections (MIP) were generated in 3 orthogonal directions.

#### CE-MRA

A three-station 3D CE-MRA was performed as previously described [29]. Prior to contrast medium administration, a non-enhanced ‘mask’ image data set was acquired with exactly the same acquisition parameters as the CE-MRA, enabling background tissue suppression by image subtraction.

#### DCE MRI

A dynamic 3D T1-weighted spoiled gradient echo sequence was used for DCE MRI with the following acquisition parameters: TR 8.0 ms, TE 0.91 ms, flip angle 30°, FOV 400 mm, matrix 128×128, NSA 1 and a parallel imaging (SENSE) reduction factor of 2 (right-left direction). Slice thickness was 6.00 mm; 12 transverse slices were imaged. Acquired voxel dimensions were 3.13×3.13×6.00 mm. Dynamic scan time was 2.6 seconds. Slices were centered at the maximum diameter of the calf. A series of 80 dynamic scans were acquired in approximately 3.5 minutes. A cuff paradigm was applied to provoke reactive hyperemia [14], [30]. A fixed dose of 2 mL gadofosveset was injected directly after cuff inflation, using an automated power injector (Medrad Spectris, Indianola, PA). Cuff inflation was directly followed by contrast injection to allow systemic contrast equilibration in the arterial blood pool during cuff compression and to ensure that DCE MRI data were acquired during the steady state, at which gadofosveset was maximally bound to albumin [27], [28], [31]. The acquisition started 330 seconds after cuff inflation. The cuff was then rapidly deflated at the start of the 6^th^ dynamic scan.

Before the cuff inflation of the dynamic sequence, a series of spoiled fast gradient echo scans with identical contrast and geometry parameters with respect to the dynamic sequence, but with varying flip angles (2, 5, 10, 15, 25 and 35°) were acquired (figure S1). These variable flip angle scans were used for T1 baseline determination (after contrast agent administration for CE-MRA) and subsequent conversion of signal changes to T1 relaxation time changes and contrast agent concentration time-courses [12], [32].

### Image Analysis

Only the most symptomatic leg was analyzed in patients, whereas for healthy control subjects the measures were determined for both legs separately and subsequently averaged. Two independent MRI readers, blinded for each other’s results and acquisition order, analyzed the data sets of healthy control subjects. Patient datasets were analyzed by one observer, foreshadowing the good interreader reproducibility we found in healthy control subjects, which were concordant to previous results [33].

The image software application MRIcro (MRIcro, http://www.mricro.com/) was used to manually draw regions of interest (ROI’s) of 3×3 pixels in the anterior tibial, gastrocnemius and soleus muscles for all 12 slices, resulting in a ROI volume of 6.3 mL. Also, a larger ROI covering the entire cross-section of the calf musculature in four successive slices was drawn. These ROI’s were subject to relatively large inter-individual differences in volume due to large variations in calf size in the subjects. An arterial ROI was drawn within the tibiofibular trunk to obtain the arterial input function (AIF). Self-written software code (Matlab, The Mathworks inc., Natick, MA) was used for the analysis of T1-weighted signal intensity time-courses for each ROI. Matlab was also used to calculate the functional microvascular parameters as represented by figure S1.

The T1-value, prior to the contrast injection of the DCE-MRI but after that of the CE-MRA, were determined from the variable flip angle series for each ROI [34]. To calculate the T1-value, the signal curve as a function of the flip angle was fitted to the signal formula for the spoiled fast gradient echo pulse sequence using a non-linear (Levenberg-Marquardt) optimization algorithm, which corrects for the inherent non-linearity in the relation between changes in MRI signal, T1, and contrast agent concentration. Any possible T2* effects were negated by the short TE used. The dynamic sequence was used to calculate the following functional measures: hyperemic fractional microvascular blood plasma volume *(V_p_,* unit: %), rate constant (*k*, unit: min^−1^) and the area-under-the-curve in the first 90 seconds after contrast arrival (AUC_90s_, unit: mM⋅s). Individual signal time-courses were normalized with respect to resting values, measured prior to contrast agent arrival and converted to contrast agent concentration, using the relation between T1 relaxation time and the signal intensity for a spoiled gradient echo pulse sequence [12].

The concentration time-course in muscle tissue 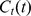
after cuff deflation was empirically modeled by the relation
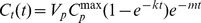
where *V_p_* is the fractional hyperemic blood plasma volume in muscle tissue, 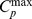
is the maximum concentration of contrast agent in blood, *k* is the rate constant describing the speed of muscle enhancement after cuff deflation, and *m* represents the tissue clearance rate. The parameters *V_p_* and *k* were calculated by non-linear curve fitting, using a Levenberg-Marquadt optimization algorithm [35]. 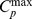
was determined from the asymptotic concentration level in the femoral artery. The concentration time-courses were used to calculate the area-under-curve for the first 90 seconds after cuff deflation (*AUC*; mMs). The functional measure *V_p_* is related to the degree of (total) microvascular dilatation, whereas the functional measures *k* and AUC reflect the rate of microvascular reactivity.

### Statistical Analysis

A two-samples *t*-test was used to determine the significance of differences in the functional parameters between patients and healthy control subjects. P<0.05 was considered statistically significant. Statistical analysis was performed with commercially available statistical software (SPSS 16.0, SPSS Inc., Chicago, IL).

For healthy control subjects the reproducibility of the measures was calculated, both at interscan and interreader level. Reproducibility of each parameter was expressed as coefficient of variation (CV in %) and the repeatability coefficient (RC). The CV is derived by dividing overall mean within-subject standard deviation (SD_ws_) by the mean measurement value over all subjects. The RC is the smallest noticeable difference that can be detected beyond measurement error and is defined as 1.96⋅√2⋅SD_ws_ [36], [37].

Assuming that interscan reproducibility was mainly influenced by scan and time related varariabilities (e.g. variations in slice positioning and day-to-day variations in physiology) it was calculated by averaging results of both MRI readers for each scan (mean of MRI 1 for reader A and B versus the mean of MRI 2 for reader A and B). Conversely, interreader reproducibility is an indicator of the image reading error (e.g. positioning and delineation of ROI’s) and is primarily influenced by reader experience. Interreader reproducibility was calculated by averaging the results of both scans for each MRI reader (mean of reader A for MRI 1 and MRI 2 versus the mean of reader B for MRI 1 and MRI 2).

## Results

All included subjects underwent DCE MRI as planned without experiencing side effects or adverse events.

### CE-MRA

CE-MRA revealed stenosis (>50%) of the SFA in 6 out of 10 patients, whereas an occlusion of the SFA was found in the remaining 4 patients. In 3 out of 10 patients there were slight vessel wall irregularities in the iliac arteries. The remaining patients had no signs of obstructive lesions in the iliac arteries. At least 2 out of 3 main arteries of the lower leg were free from obstructive lesions in all patients. CE-MRA revealed no signs of PAD in any of the control subjects.

### DCE MRI

Examples of DCE MRI derived time-courses of the entire cross-section of the calf musculature and arterial blood are presented in figure S2. Muscle tissue enhancement was generally less strong in patients compared to healthy control subjects (figure S2). Signal time-courses of the individual muscle groups showed similar results. Strongest enhancement was found in the soleus muscle.

An AIF was obtained for each individual subject within the most prominent arterial structure visible, which was either the tibiofibular trunk or posterior tibial artery in most patients. Quality of the AIF was lower in patients in comparison with the AIF of healthy control subjects. There was a trend (p = 0.07) towards higher maximum concentration of the AIF in patients compared to the healthy control subjects.

### Patients Versus Healthy Control Subjects

Table S1 lists the values of DCE MRI measures obtained in patients and healthy control subjects. *V_p_* was significantly lower for all muscle groups in PAD patients compared to healthy control subjects. Differences in *V_p_* between patients and controls ranged from 3.2–4.8% (p<0.01). For both patients and controls the differences in *V_p_* between muscle groups were not significant (p = 0.49 and p = 0.15, respectively). *V_p_* of the entire cross-section of the calf musculature was significantly higher compared to the *V_p_* of individual muscle groups, both for patients and healthy control subjects (p<0.01). The rate constant *k* was significantly lower in PAD patients for all muscle groups (range differences 5.5–7.9 min^−1^, p<0.01). The AUC only was significantly lower in PAD patients for the anterior tibial muscle (p<0.01). *V_p_, k* or AUC were not consistently higher or lower in PAD patients with stenosis (n  = 6) compared to PAD patients with an occlusion (n = 4) of the SFA.

### Reproducibility

Values and reproducibility measures of the pre-contrast T1 assessment in healthy control subjects are given in table S2. T1 assessment showed interscan and interreader CV’s below 6 and 7% respectively.

Reproducibility of the DCE MRI measurements in healthy control subjects is listed in table S3. CV and RC of *V_p_* for the different muscle groups were below 17% and 6%, respectively, at interscan level and below 16% and 6%, respectively, at interreader level. Interreader and interscan reproducibility of *V_p_* were comparable for most muscle groups. Reproducibility values of *V_p_* were comparable for the different muscles. Interscan reproducibility of *k* and AUC showed CV values as high as 49% and 37%, respectively. Interreader reproducibility was markedly better with a maximum CV of 22% and 27% for *k* and AUC, respectively. Interreader CV was lowest for the entire muscle group (8.4% and 1.8% for *k* and AUC, respectively).

## Discussion

In this study we found that gadofosveset, a reversible albumin binding (blood pool) contrast agent, can be used in DCE MRI to reproducibly determine a functional surrogate of the hyperemic microvascular blood plasma volume in the calf. Using this functional measure, the fractional microvascular blood plasma volume *V_p_*, patients with PAD revealed a reduced *V_p_* for all muscle types in the calf relative to healthy controls. Moreover, patients with PAD could be discriminated from healthy subjects on the basis of *V_p_*. Measures that quantified the rate of vasodilation (k and AUC) were also reduced in patients with PAD compared to healthy controls. However, interscan and interreader reproducibility of *V_p_* were better compared to those of measures that reflect the vasodilation rate (*k* and AUC). We also found that functional DCE MRI with a blood pool contrast agent can easily be combined with clinical CE-MRA to assess both macrovascular morphology and microvascular functionality in patients with PAD.

### Pathophysiologic Effect in PAD

The fractional microvascular blood plasma volume *V_p_* of the calf musculature in PAD patients with intermittent claudication was significantly lower than in healthy control subjects. A lower *V_p_* in patients with PAD can possibly be explained by impaired microvascular dilatative capacity, which is the result of endothelial dysfunction [38], [39], [40], [41], [42], [43]. This means that DCE MRI can be used to determine the fractional microvascular blood plasma volume of each large muscle group of the calf and as such can identify patients with reduced *V_p_* as compared to healthy controls.

*V_p_* did show consistent differences between PAD patients with stenosis or occlusions. This can be explained partly by the small sample size (respectively six PAD patients with stenosis and four with occlusion of the SFA), but also by the fact that most of the patients with an occlusion of the SFA revealed well developed collateral arteries at CE-MRA and both patients with stenosis and occlusion had the same clinical symptoms (i.e. intermittent claudication).

There were no significant differences between individual muscle groups within patients or controls. This indicates that this method does not distinguish different muscles of the calf. In healthy control subjects, *V_p_* of the entire cross-section of the calf musculature showed significantly higher values when compared to *V_p_* of the individual muscles. In patients a similar trend was observed. A higher *V_p_* for the entire cross-section of the calf musculature can be explained by the inclusion of relatively large arterial and venous structures (in comparison to microvessels) in these regions of interest, increasing the average amount of contrast agent within the ROI which results in a higher *V_p_*. To avoid relatively large blood vessels, assessment of *V_p_* of individual muscles is preferred over the *V_p_* of the entire cross-section of the calf musculature.

The measures *k* and the *AUC* both reflect the initial speed of signal enhancement during reactive hyperemia and were lower in patients compared to healthy controls. This finding indicates lower vascular reactivity in patients with PAD, which is in line with the results of previous studies [14], [19].

### Reproducibility

Interscan reproducibility of *V_p_* in DCE MRI was good compared to *k* and the *AUC*. The interscan CV in healthy control subjects of all muscle groups was lower than the relative difference between patients and healthy control subjects. This implies that the reproducibility of the measurement is sufficient to discriminate patients with PAD from healthy controls. The RC for the anterior tibial and gastrocnemius muscles was lower compared to the absolute difference between patients and healthy controls. The RC for the entire cross-section of the calf musculature and the soleus muscle was comparable to the absolute difference between patients and healthy control subjects. A poor RC is probably the result of our small study population and these results might improve with a larger group of subjects.

Reproducibility of *V_p_* was in general much higher than the reproducibility of *k* and the *AUC*, both at interscan and interreader level. This is clearly reflected by the large RC values for *k* and AUC, which are comparable or even higher than the measured values for *k* and AUC in healthy control subjects. These findings are in line with those found by Galbraith et al. concerning the extravasation rate constant *K^trans^*, using low molecular weight (i.e. small-sized) extracellular contrast agents [19]. For a blood pool acting contrast agent, *k* mainly represents the speed of the filling of the microvasculature in the time period of the measurement, rather than the extravasation of contrast agent into the interstitial space. This rapid filling phase did not prove reproducible and is therefore not suitable as a monitoring measure in clinical practice.

T1 mapping showed good interscan and interreader reproducibility of T1 values for each muscle group. High interreader reproducibility indicates that the technique is relative insensitive for the exact location at which a ROI is drawn within a muscle group and that the technique is highly reader-independent. This is especially desirable for monitoring purposes, as in clinical routine it is likely that follow-up examinations will be assessed by different readers. Good reproducibility of T1 mapping suggests that our scan and analysis method is reliable and that the moderate reproducibility we found for *V_p_* is the result of biologic factors and/or the cuff paradigm.

### Clinical Relevance

Functional assessment of muscle perfusion of the lower extremities with dynamic contrast-enhanced (DCE) MRI, could be a valuable addition to the diagnostic work-up in PAD to objectively determine disease severity. An added advantage is that DCE MRI can be combined with the standard morphological assessment of PAD with CE-MRA. Also, DCE-MRI could be of value in non-invasive assessment of the efficacy of novel therapeutic strategies such as stem cell and gene therapy. DCE MRI of calf musculature may indicate the contribution of macrovascular blood flow on muscle perfusion and provide an objective diagnostic tool to determine the influence and severity of microvascular dysfunction in PAD [13], [14], [15], [19].

Commonly used extracellular contrast agents rapidly extravasate into the interstitial space and are therefore less suitable to determine biomarkers of the microvascular blood plasma volume [25].

### Study Considerations

Although we found large differences between patients with PAD and healthy control subjects for *V_p_* and the reproducibility of *V_p_* was good, the number of subjects in this study is relatively small and further research will be needed to confirm our results by blinded analysis in more patients and compare them with age and gender matched control subjects. The observed differences between patients with PAD and the controls is expectedly smaller for age-matched, thus older, controls. In addition, it would be clinically relevant to include patients with different symptoms of PAD to assess variations in the involvement of microvascular impairment in PAD.

In all subjects a three-station CE-MRA of the peripheral arteries was performed, prior to the DCE MRI. For CE-MRA the first bolus injection of gadofosveset was used. This means all subjects already had gadofosveset in their systemic circulation at the start of the DCE MRI. The residual presence of contrast agent in the circulation affects the T1 determination and its variability. However, a combination of both exams is highly desirable for clinical practice and our results prove the possibility to discriminate patients from control subjects and provided good reproducibility of *V_p_* (and T1), despite the previous bolus injection for angiography.

Although in reality a small fraction (±15%) of unbound gadosfosveset is available for extravasation into the interstitial space, the influence of this fraction upon the outcome can be neglected as the relative relaxivity of albumin-bound gadofosveset is much higher compared to the unbound fraction (r_1_ of respectively 19 and 5.2 L·mmol^−1^·s^−1^ in blood plasma at 37°C at 1.5 T) and the total fraction of albumin-bond gadofosveset (±85%) is much larger as compared to the unbound fraction [27], [28], [31]. In this study, gadofosveset proved to be a usable contrast agent for DCE MRI of muscle tissue in PAD to obtain a surrogate functional measure of microvascular blood plasma volume in patients with PAD.

### Conclusion

DCE MRI after CE-MRA using a blood pool contrast agent is able to determine the hyperemic microvascular blood plasma volume *V_p_* of the calf musculature and to identify patients with PAD as compared to healthy control subjects. In addition, the reproducibility of *V_p_* was good in healthy control subjects. Together with the ability to acquire DCE MRI data combined with CE-MRA during a single examination makes DCE MRI with a blood pool acting agent a valuable addition to CE-MRA to assess both the macrovascular morphology and functional (hemodynamic) status of the calf microvasculature in patients with PAD.

## Supporting Information

Figure S1 Overview of the imaging protocol. DCE MRI was preceded by conventional three-station contrast-enhanced MR angiography (CE-MRA) for which a dose of 8 mL gadofosveset was administered. CE-MRA was followed by ‘pre-contrast’ T1 determination. A 6-minute cuff compression of the thigh was used to provoke reactive hyperemia within the calf musculature. Directly after cuff inflation a single dose of 2 mL gadofosveset was injected. Half a minute before cuff deflation the dynamic contrast-enhanced (DCE) MRI was started.
(TIF)

Figure S2 Coronal maximum intensity projection (MIP) of a CE-MRA examination of the peripheral arterial tree of a patient with PAD (left panel) and examples of axial cross-sectional CE-MRA and DCE images of a patient with PAD (center panel) and a healthy control subject (right panel), respectively. The comparison for DCE MRI between patients with PAD and healthy control subjects was made for the tibial anterior (A), gastrocnemius (B) and soleus (C) muscle, as well as the entire cross-section of the calf musculature (area within the black dotted lines). The lower center and right panels show representative examples of the relative signal change in blood and the entire cross-section of the calf musculature before and after cuff release in a patient with PAD and healthy control, respectively.
(TIF)

Table S1 DCE MRI of the calf musculature in patients with PAD and healthy control subjects Caption: values are represented as mean ± SD; V_p_, fractional microvascular blood plasma volume; k, rate constant; AUC_90s_, area under the curve for the first 90 seconds after cuff release. ^*^p<0.01, ^**^ p<0.05.
(DOCX)

Table S2 Reproducibility of T1 determination in healthy control subjects Caption: values are presented as mean ± SD; T_1pre_,T_1_ before administration of contrast agent; CV, coefficient of variation; RC, repeatability coefficient. ^*^Pre-contrast T1 values were actually obtained after a prior injection of 8 mL gadofosveset, as used for contrast-enhanced MR angiography of the lower extremities.
(DOCX)

Table S3 Reproducibility of DCE MRI in healthy control subjects Caption: values are presented as mean ± SD; A, maximum concentration; V_p_, fractional microvascular blood plasma volume; k, rate constant; AUC_90s_, area under the curve for the first 90 seconds; CV, coefficient of variation; RC, repeatability coefficient.
(DOCX)

## References

[1] Hirsch AT, Haskal ZJ, Hertzer NR, Bakal CW, Creager MA (2006). ACC/AHA 2005 Practice Guidelines for the management of patients with peripheral arterial disease (lower extremity, renal, mesenteric, and abdominal aortic): a collaborative report from the American Association for Vascular Surgery/Society for Vascular Surgery, Society for Cardiovascular Angiography and Interventions, Society for Vascular Medicine and Biology, Society of Interventional Radiology, and the ACC/AHA Task Force on Practice Guidelines (Writing Committee to Develop Guidelines for the Management of Patients With Peripheral Arterial Disease): endorsed by the American Association of Cardiovascular and Pulmonary Rehabilitation; National Heart, Lung, and Blood Institute; Society for Vascular Nursing; TransAtlantic Inter-Society Consensus; and Vascular Disease Foundation.. Circulation.

[2] Norgren L, Hiatt WR, Dormandy JA, Nehler MR, Harris KA (2007). Inter-Society Consensus for the Management of Peripheral Arterial Disease (TASC II)..

[3] Gerhard-Herman M, Gardin JM, Jaff M, Mohler E, Roman M (2006). Guidelines for noninvasive vascular laboratory testing: a report from the American Society of Echocardiography and the Society for Vascular Medicine and Biology.. Vasc Med.

[4] Creager MA (1997). Clinical assessment of the patient with claudication: the role of the vascular laboratory.. Vasc Med.

[5] Aslam F, Haque A, Foody J, Lee LV (2009). Peripheral arterial disease: current perspectives and new trends in management.. South Med J.

[6] McDermott MM, Criqui MH, Greenland P, Guralnik JM, Liu K (2004). Leg strength in peripheral arterial disease: associations with disease severity and lower-extremity performance.. J Vasc Surg.

[7] McDermott MM, Greenland P, Liu K, Guralnik JM, Criqui MH (2001). Leg symptoms in peripheral arterial disease: associated clinical characteristics and functional impairment.. JAMA.

[8] Resnick HE, Lindsay RS, McDermott MM, Devereux RB, Jones KL (2004). Relationship of high and low ankle brachial index to all-cause and cardiovascular disease mortality: the Strong Heart Study.. Circulation.

[9] Begelman SM, Jaff MR (2006). Noninvasive diagnostic strategies for peripheral arterial disease.. Cleve Clin J Med.

[10] Chao CY, Cheing GL (2009). Microvascular dysfunction in diabetic foot disease and ulceration.. Diabetes Metab Res Rev.

[11] Wong WT, Wong SL, Tian XY, Huang Y (2010). Endothelial dysfunction: the common consequence in diabetes and hypertension.. J Cardiovasc Pharmacol.

[12] de Lussanet QG, van Golde JC, Beets-Tan RG, Post MJ, Huijberts MS (2007). Dynamic contrast-enhanced MRI of muscle perfusion combined with MR angiography of collateral artery growth in a femoral artery ligation model.. NMR Biomed.

[13] Isbell DC, Epstein FH, Zhong X, DiMaria JM, Berr SS (2007). Calf muscle perfusion at peak exercise in peripheral arterial disease: measurement by first-pass contrast-enhanced magnetic resonance imaging.. J Magn Reson Imaging.

[14] Thompson RB, Aviles RJ, Faranesh AZ, Raman VK, Wright V (2005). Measurement of skeletal muscle perfusion during postischemic reactive hyperemia using contrast-enhanced MRI with a step-input function.. Magn Reson Med.

[15] Weber MA, Krix M, Delorme S (2007). Quantitative evaluation of muscle perfusion with CEUS and with MR.. Eur Radiol.

[16] Nelemans PJ, Leiner T, de Vet HC, van Engelshoven JM (2000). Peripheral arterial disease: meta-analysis of the diagnostic performance of MR angiography.. Radiology.

[17] Koelemay MJ, Lijmer JG, Stoker J, Legemate DA, Bossuyt PM (2001). Magnetic resonance angiography for the evaluation of lower extremity arterial disease: a meta-analysis.. JAMA.

[18] Menke J, Larsen J (2010). Meta-analysis: Accuracy of contrast-enhanced magnetic resonance angiography for assessing steno-occlusions in peripheral arterial disease.. Ann Intern Med.

[19] Galbraith SM, Lodge MA, Taylor NJ, Rustin GJ, Bentzen S (2002). Reproducibility of dynamic contrast-enhanced MRI in human muscle and tumours: comparison of quantitative and semi-quantitative analysis.. NMR Biomed.

[20] de Vries M, de Koning PJ, de Haan MW, Kessels AG, Nelemans PJ (2005). Accuracy of semiautomated analysis of 3D contrast-enhanced magnetic resonance angiography for detection and quantification of aortoiliac stenoses.. Invest Radiol.

[21] Leiner T, Kessels AG, Nelemans PJ, Vasbinder GB, de Haan MW (2005). Peripheral arterial disease: comparison of color duplex US and contrast-enhanced MR angiography for diagnosis.. Radiology.

[22] Tofts PS, Brix G, Buckley DL, Evelhoch JL, Henderson E (1999). Estimating kinetic parameters from dynamic contrast-enhanced T(1)-weighted MRI of a diffusable tracer: standardized quantities and symbols.. J Magn Reson Imaging.

[23] Kershaw LE, Buckley DL (2006). Precision in measurements of perfusion and microvascular permeability with T1-weighted dynamic contrast-enhanced MRI.. Magn Reson Med.

[24] Mitchell GF (2008). Effects of central arterial aging on the structure and function of the peripheral vasculature: implications for end-organ damage.. J Appl Physiol.

[25] Tofts PS (1997). Modeling tracer kinetics in dynamic Gd-DTPA MR imaging.. J Magn Reson Imaging.

[26] Tofts PS, Berkowitz BA (1994). Measurement of capillary permeability from the Gd enhancement curve: a comparison of bolus and constant infusion injection methods.. Magn Reson Imaging.

[27] Caravan P, Cloutier NJ, Greenfield MT, McDermid SA, Dunham SU (2002). The interaction of MS-325 with human serum albumin and its effect on proton relaxation rates.. J Am Chem Soc.

[28] Caravan P, Parigi G, Chasse JM, Cloutier NJ, Ellison JJ (2007). Albumin binding, relaxivity, and water exchange kinetics of the diastereoisomers of MS-325, a gadolinium(III)-based magnetic resonance angiography contrast agent.. Inorg Chem.

[29] de Vries M, Nijenhuis RJ, Hoogeveen RM, de Haan MW, van Engelshoven JM (2005). Contrast-enhanced peripheral MR angiography using SENSE in multiple stations: feasibility study.. J Magn Reson Imaging.

[30] Ledermann HP, Schulte AC, Heidecker HG, Aschwanden M, Jager KA (2006). Blood oxygenation level-dependent magnetic resonance imaging of the skeletal muscle in patients with peripheral arterial occlusive disease.. Circulation.

[31] Rohrer M, Bauer H, Mintorovitch J, Requardt M, Weinmann HJ (2005). Comparison of magnetic properties of MRI contrast media solutions at different magnetic field strengths.. Invest Radiol.

[32] Haacke M, Brown R, Thompson M, Venkatesan R, Haacke M, Brown R, Thompson M, Venkatesan R (1999). T1 estimation from SSI measurements at multiple flip angles.. Magnetic resonance imaging: physical principles ans sequence design.

[33] Versluis B, Backes WH, van Eupen MG, Jaspers K, Nelemans PJ (2011). Magnetic resonance imaging in peripheral arterial disease: reproducibility of the assessment of morphological and functional vascular status.. Invest Radiol.

[34] Haacke EM, Filleti CL, Gattu R, Ciulla C, Al-Bashir A (2007). New algorithm for quantifying vascular changes in dynamic contrast-enhanced MRI independent of absolute T1 values.. Magn Reson Med.

[35] Press WH (1992). Numerical recipes in C : the art of scientific computing. Cambridge; New York: Cambridge University Press..

[36] Bland JM, Altman DG (1996). Measurement error.. BMJ.

[37] Jansen JF, Kooi ME, Kessels AG, Nicolay K, Backes WH (2007). Reproducibility of quantitative cerebral T2 relaxometry, diffusion tensor imaging, and 1H magnetic resonance spectroscopy at 3.0 Tesla.. Invest Radiol.

[38] Grover-Paez F, Zavalza-Gomez AB (2009). Endothelial dysfunction and cardiovascular risk factors.. Diabetes Res Clin Pract.

[39] Libby P, Ridker PM, Hansson GK (2009). Inflammation in atherosclerosis: from pathophysiology to practice.. J Am Coll Cardiol.

[40] Rocha VZ, Libby P (2009). Obesity, inflammation, and atherosclerosis.. Nat Rev Cardiol.

[41] Ribeiro F, Alves AJ, Teixeira M, Ribeiro V, Duarte JA (2009). Endothelial function and atherosclerosis: circulatory markers with clinical usefulness.. Rev Port Cardiol.

[42] Vita JA, Hamburg NM (2010). Does endothelial dysfunction contribute to the clinical status of patients with peripheral arterial disease?.

[43] Ziegler MA, Distasi MR, Bills RG, Miller SJ, Alloosh M (2010). Marvels, mysteries, and misconceptions of vascular compensation to peripheral artery occlusion.. Microcirculation.

