# Supplementary figures and images for: Dynamic Contrast-Enhanced MRI Assessment of Hyperemic Fractional Microvascular Blood Plasma Volume in Peripheral Arterial Disease: Initial Findings
Source: PLoS One. 2012 May 25;7(5):e37756. doi: 10.1371/journal.pone.0037756 (PMC3360623; doi:10.1371/journal.pone.0037756)

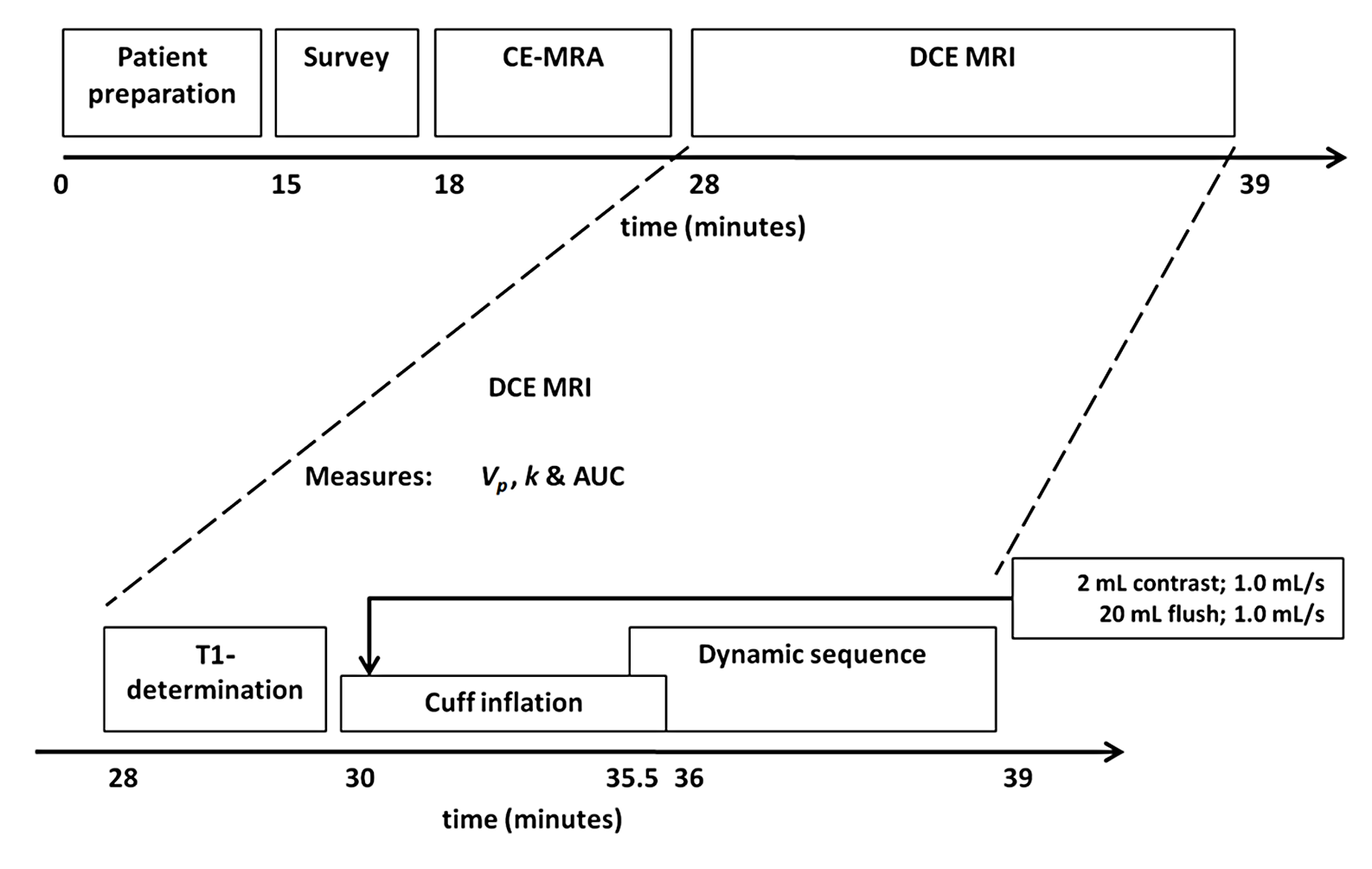

Supplement: Figure S1 — Overview of the imaging protocol. DCE MRI was preceded by conventional three-station contrast-enhanced MR angiography (CE-MRA) for which a dose of 8 mL gadofosveset was administered. CE-MRA was followed by ‘pre-contrast’ T1 determination. A 6-minute cuff compression of the thigh was used to provoke reactive hyperemia within the calf musculature. Directly after cuff inflation a single dose of 2 mL gadofosveset was injected. Half a minute before cuff deflation the dynamic contrast-enhanced (DCE) MRI was started. (TIF) [file pone.0037756.s001.tif]

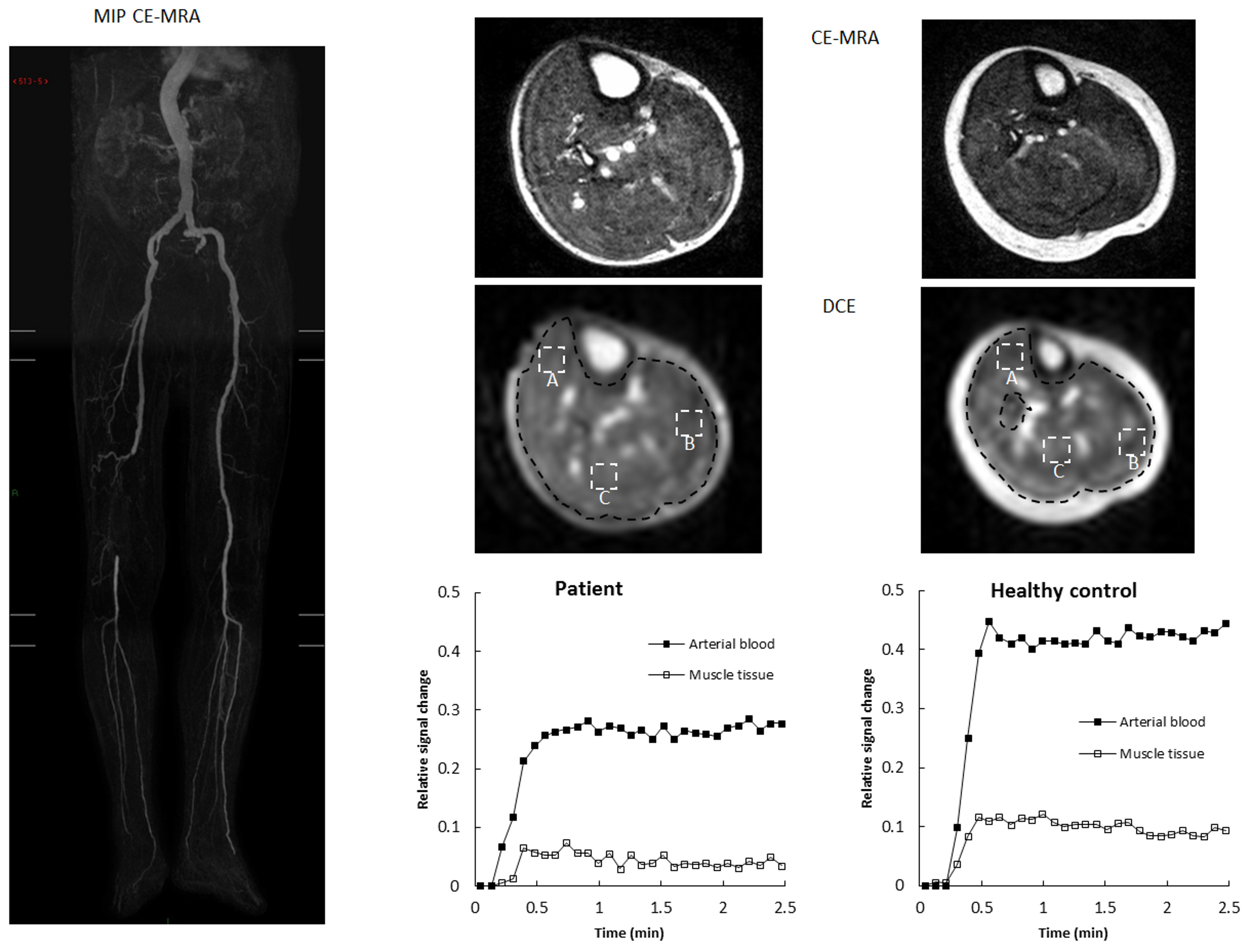

Supplement: Figure S2 — Coronal maximum intensity projection (MIP) of a CE-MRA examination of the peripheral arterial tree of a patient with PAD (left panel) and examples of axial cross-sectional CE-MRA and DCE images of a patient with PAD (center panel) and a healthy control subject (right panel), respectively. The comparison for DCE MRI between patients with PAD and healthy control subjects was made for the tibial anterior (A), gastrocnemius (B) and soleus (C) muscle, as well as the entire cross-section of the calf musculature (area within the black dotted lines). The lower center and right panels show representative examples of the relative signal change in blood and the entire cross-section of the calf musculature before and after cuff release in a patient with PAD and healthy control, respectively. (TIF) [file pone.0037756.s002.tif]
