# Supplementary material for: Dynamic Contrast-Enhanced MRI Assessment of Hyperemic Fractional Microvascular Blood Plasma Volume in Peripheral Arterial Disease: Initial Findings
Source: PLoS One. 2012 May 25;7(5):e37756. doi: 10.1371/journal.pone.0037756 (PMC3360623; doi:10.1371/journal.pone.0037756)
Supplement: Table S1 — DCE MRI of the calf musculature in patients with PAD and healthy control subjects Caption: values are represented as mean ± SD; Vp, fractional microvascular blood plasma volume; k, rate constant; AUC90s, area under the curve for the first 90 seconds after cuff release. *p<0.01, ** p<0.05. (DOCX) [file pone.0037756.s003.docx]

|  | | |  |  | **Patients** | **Healthy controls** |  |
| --- | --- | --- | --- | --- | --- | --- | --- |
|  | | |  |  | (n = 10) | (n = 10) |  |
| **Anterior tibial muscle** | | | | |  |  |  |
|  | *V_p_* | % | | | 4.3 ± 1.6 | 9.1 ± 2.0^*^ |  |
|  | *K* | min^-1^ | | | 2.3 ± 1.6 | 11.2 ± 5.6^*^ |  |
|  | *AUC_90s_* | mMs | | | 0.35 ± 0.22 | 0.64 ± 0.19^*^ |  |
| **Gastrocnemius muscle** | | | | |  |  |  |
|  | *V_p_* | % | | | 5.0 ± 3.3 | 8.9 ± 1.9^*^ |  |
|  | *K* | min^-1^ | | | 4.1 ± 4.1 | 9.6 ± 4.0^*^ |  |
|  | *AUC_90s_* | mMs | | | 0.49 ± 0.26 | 0.55 ± 0.14 | (p = 0.50) |
| **Soleus muscle** | | | | |  |  |  |
|  | *V_p_* | % | | | 6.1 ± 3.6 | 9.3 ± 2.1^**^ |  |
|  | *K* | min^-1^ | | | 5.1 ± 3.7 | 12.3 ± 4.1^*^ |  |
|  | *AUC_90s_* | mMs | | | 0.64 ± 0.28 | 0.65 ± 0.15 | (p = 0.90) |
| **Cross-section calf musculature** | | | | |  |  |  |
|  | *V_p_* | % | | | 7.2 ± 4.3 | 12.0 ± 2.4^*^ |  |
|  | *k* | min^-1^ | | | 5.5 ± 3.6 | 10.4 ± 4.1^*^ |  |
|  | *AUC_90s_* | mMs | | | 0.78 ± 0.17 | 0.87 ± 0.17 | (p = 0.18) |
