# Supplementary material for: Dynamic Contrast-Enhanced MRI Assessment of Hyperemic Fractional Microvascular Blood Plasma Volume in Peripheral Arterial Disease: Initial Findings
Source: PLoS One. 2012 May 25;7(5):e37756. doi: 10.1371/journal.pone.0037756 (PMC3360623; doi:10.1371/journal.pone.0037756)
Supplement: Table S2 — Reproducibility of T1 determination in healthy control subjects Caption: values are presented as mean ± SD; T1pre,T1 before administration of contrast agent; CV, coefficient of variation; RC, repeatability coefficient. *Pre-contrast T1 values were actually obtained after a prior injection of 8 mL gadofosveset, as used for contrast-enhanced MR angiography of the lower extremities. (DOCX) [file pone.0037756.s004.docx]

|  | | |  |  | **T_1pre_^*^** |
| --- | --- | --- | --- | --- | --- |
|  | | |  |  |  |
| **Anterior tibial muscle** | | | | | 688 ± 29 ms |
|  | | | | |  |
| Interscan | | | | |  |
|  |  | CV | | | 3.4 % |
|  |  | RC | | | 63 ms |
| Interreader | | | | |  |
|  |  | CV | | | 1.2 % |
|  |  | RC | | | 22 ms |
| **Gastrocnemius muscle** | | | | | 722 ± 56 ms |
|  | | | | |  |
| Interscan | | | | |  |
|  |  | CV | | | 5.4 % |
|  |  | RC | | | 108 ms |
| Interreader | | | | |  |
|  |  | CV | | | 6.4 % |
|  |  | RC | | | 131 ms |
| **Soleus muscle** | | | | | 667 ± 32 ms |
|  | | | | |  |
| Interscan | | | | |  |
|  |  | CV | | | 5.0 % |
|  |  | RC | | | 91 ms |
| Interreader | | | | |  |
|  |  | CV | | | 5.9 % |
|  |  | RC | | | 109 ms |
| **Cross-section calf musculature** | | | | | 625 ± 28 ms |
|  | | | | |  |
| Interscan | | | | |  |
|  |  | CV | | | 4.9 % |
|  |  | RC | | | 84 ms |
| Interreader | | | | |  |
|  |  | CV | | | 2.6 % |
|  |  | RC | | | 44 ms |
