# Supplementary material for: Dynamic Contrast-Enhanced MRI Assessment of Hyperemic Fractional Microvascular Blood Plasma Volume in Peripheral Arterial Disease: Initial Findings
Source: PLoS One. 2012 May 25;7(5):e37756. doi: 10.1371/journal.pone.0037756 (PMC3360623; doi:10.1371/journal.pone.0037756)
Supplement: Table S3 — Reproducibility of DCE MRI in healthy control subjects Caption: values are presented as mean ± SD; A, maximum concentration; Vp, fractional microvascular blood plasma volume; k, rate constant; AUC90s, area under the curve for the first 90 seconds; CV, coefficient of variation; RC, repeatability coefficient. (DOCX) [file pone.0037756.s005.docx]

|  | | |  |  | ***V_p_*** | ***k*** | ***AUC_90s_*** |
| --- | --- | --- | --- | --- | --- | --- | --- |
|  | | |  |  |  |  |  |
| **Anterior tibial muscle** | | | | | 9.1 ± 2.0 % | 11.2 ± 5.6 min^-1^ | 0.64 ± 0.19 mMs |
|  | | | | |  |  |  |
| Interscan | | | | |  |  |  |
|  |  | CV | | | 12.5 % | 48.8 % | 36.5 % |
|  |  | RC | | | 3.1 % | 15.1 min^-1^ | 0.64 mMs |
| Interreader | | | | |  |  |  |
|  |  | CV | | | 13.0 % | 22.8 % | 16.2% |
|  |  | RC | | | 3.3 % | 7.1 min^-1^ | 0.29 mMs |
| **Gastrocnemius muscle** | | | | | 8.9 ± 1.9 % | 9.6 ± 4.0 min^-1^ | 0.55 ± 0.14 mMs |
|  | | | | |  |  |  |
| Interscan | | | | |  |  |  |
|  |  | CV | | | 9.7 % | 37.8 % | 25.5 % |
|  |  | RC | | | 2.4 % | 10.0 min^-1^ | 0.39 mMs |
| Interreader | | | | |  |  |  |
|  |  | CV | | | 16.0 % | 22.1 % | 27.3 % |
|  |  | RC | | | 3.9 % | 5.9 min^-1^ | 0.41 mMs |
| **Soleus muscle** | | | | | 9.3 ± 2.1 % | 12.3 ± 4.1 min^-1^ | 0.65 ± 0.15 mMs |
|  | | | | |  |  |  |
| Interscan | | | | |  |  |  |
|  |  | CV | | | 13.3 % | 19.9 % | 23.3 % |
|  |  | RC | | | 3.4 % | 6.8 min^-1^ | 0.42 mMs |
| Interreader | | | | |  |  |  |
|  |  | CV | | | 11.2 % | 18.7 % | 12.0 % |
|  |  | RC | | | 2.9 % | 6.3 min^-1^ | 0.22 mMs |
| **Cross-section calf musculature** | | | | | 12.0 ± 2.4 % | 10.4 ± 4.1 min^-1^ | 0.87 ± 0.17 mMs |
|  | | | | |  |  |  |
| Interscan | | | | |  |  |  |
|  |  | CV | | | 16.3 % | 34.8 % | 20.3 % |
|  |  | RC | | | 5.4 % | 9.9 min^-1^ | 0.49 mMs |
| Interreader | | | | |  |  |  |
|  |  | CV | | | 2.1 % | 8.4 % | 1.8 % |
|  |  | RC | | | 0.7 % | 2.4 min^-1^ | 0.04 mMs |
